# Single Molecule Spectroscopy of Monomeric LHCII: Experiment and Theory

**DOI:** 10.1038/srep26230

**Published:** 2016-05-18

**Authors:** Pavel Malý, J. Michael Gruber, Rienk van Grondelle, Tomáš Mančal

**Affiliations:** 1 Department of Physics and Astronomy, Faculty of Sciences, Vrije Universiteit Amsterdam, 1081 HV Amsterdam, The Netherlands; 2 Institute of Physics, Faculty of Mathematics and Physics, Charles University in Prague, Ke Karlovu 5, CZ-121 16 Prague 2, Czech Republic

## Abstract

We derive approximate equations of motion for excited state dynamics of a multilevel open quantum system weakly interacting with light to describe fluorescence-detected single molecule spectra. Based on the Frenkel exciton theory, we construct a model for the chlorophyll part of the LHCII complex of higher plants and its interaction with previously proposed excitation quencher in the form of the lutein molecule Lut 1. The resulting description is valid over a broad range of timescales relevant for single molecule spectroscopy, i.e. from ps to minutes. Validity of these equations is demonstrated by comparing simulations of ensemble and single-molecule spectra of monomeric LHCII with experiments. Using a conformational change of the LHCII protein as a switching mechanism, the intensity and spectral time traces of individual LHCII complexes are simulated, and the experimental statistical distributions are reproduced. Based on our model, it is shown that with reasonable assumptions about its interaction with chlorophylls, Lut 1 can act as an efficient fluorescence quencher in LHCII.

Photosynthesis, arguably the most important photo-induced process on Earth, converts the energy of light into its chemically/biologically useful form. It is often argued that this conversion is remarkably efficient. However, it has to be distinguished between the *quantum* efficiency, reaching almost unity^1^,^2^, and light-to-chemical energy efficiency, which is significantly lower, in the order of several percent^3^. This is given by an evolutionary pressure on the development of a robust photosynthetic machinery optimized for survival rather than energy conversion efficiency. Energy relaxation processes are integral part of the photosynthetic function as they enable the energy transfer to proceed unidirectionally^4^, and provide protection of the photosynthetic apparatus against harmful over-excitation. The photosynthetic machinery of plants has developed a complex hierarchy of self-regulatory mechanisms to avoid excess excitation or (when unavoidable) to dissipate it into heat^5^. Starting from processes controlled on the macroscopic level (e.g. orientation of leaves), over spontaneous microscopic (cellular) events such as chloroplast movements, to truly nano- and sub-nanoscopic mechanisms such as reorganization of antenna complexes and direct regulation of energy transfer on the level of small groups of interacting chromophores, plants actively react to changing illumination conditions. The sub-nanoscopic processes, which are the focus of the present study, operate in response to the increase of ΔpH across the thylakoid membrane. Such an increase is an indicator of high illumination. Nowadays it is generally accepted that carotenoids are involved in these energy dissipation processes. The precise molecular mechanism is, however, still subject of discussion^6^,^7^,^8^,^9^,^10^,^11^,^12^,^13^. It is likely that different mechanisms evolved in different classes of organisms and/or that several mechanisms operate at once.

Most of our current knowledge about the early processes of photosynthesis was obtained by ultrafast spectroscopy. While conventional bulk spectroscopies are extremely useful in following ultrafast photo-induced events in photosynthesis, whenever structural inhomogeneity of the sample is involved, the information obtained from these spectroscopies becomes obscured by an inevitable ensemble averaging. Some well established spectroscopic methods, such as hole-burning^14^,^15^,^16^, and some modern multidimensional methods, such as coherent two-dimensional spectroscopy^17^, provide certain access to the homogeneous properties. Information on the behaviour of individual molecules has been, however, available only since the advent of single-molecule spectroscopy (SMS)^18^.

The system studied in this work, the light harvesting complex II (LHCII) of higher plants, is the plants’ major light-harvesting antenna containing almost half of all the chlorophyll in the chloroplast. Correspondingly, most of the light absorption and subsequent energy transfer processes in plants and algae occur in this complex. The LHCII antenna occurs naturally in a trimeric form and its main function is to deliver excitation energy to the nearby photosystem II (PSII). Given the major role of LHCII in light-harvesting and energy transfer, it is not surprising that it is also implicated in participating in regulated energy dissipation, the so-called non-photochemical quenching (NPQ)^7^,^9^,^19^. The crystal structure of the complex^20^ enables us efficient theoretical modeling of the complex’s spectroscopic properties using its chromophores (chlorophylls (Chl) and carotenoids) as the model units. The model parameters such as electronic coupling are greatly constrained by the known mutual orientation and distances. Existence of a large body of previous measurements together with theoretical attempts to fit this whole body of data to a single model^21^,^22^,^23^ gives a great confidence in extending the modeling towards single molecular experiments.

In recent years, single-molecule spectroscopy (SMS) experiments on several photosynthetic antenna complexes including the LHCII were performed. Fluorescence spectral peak distributions^24^, spectral diffusion^25^, fluorescence intensity distributions^26^,^27^ and time traces^28^ were obtained from these measurements. In many cases also fluorescence intermittency (blinking) was observed, and it was conjectured that in the case of LHCII the process behind the fluorescence intermittency plays a role in NPQ^26^.

As far as the theory of the SMS of LHCII is concerned, the ensemble-averaged spectra and also the peak distributions of LHCII trimers can be successfully explained by the disordered excitonic model^24^. In general, the excitonic model was successfully applied in the past on fs to ps timescale, and it represents an indispensable tool in analyzing ultrafast spectroscopic experiments on molecular aggregates and in particular on photosynthetic antennae^4^. Its application to longer time scales of seconds and minutes is conditioned by the assumption of a certain separation of time-scales. Over the course of the excitation-emission cycle (nanoseconds), individual chromophores of a complex are assumed to be found in fixed spatial arrangements and experiencing an environment described by a fixed set of parameters. The emission spectrum of a complex in such a fixed spatial arrangement is predicted by the excitonic model which gives the population distribution of the excited state manifold and the probability of emission at the corresponding transition frequencies. The spectrum of exciting light matters only to the extent to which the excited states reached at a given excitation wavelength are connected to the final state by some relaxation pathways. Once the pathways are available, the final state is given irrespective of the initial state after absorption of light. Because only the final distribution of excited state population matters, the changes (switches) of the spatial arrangement or environmental conditions occurring on the sub-nanosecond time scale are only observed as sudden changes (with respect to the nanosecond fluorescence time scale) of the fluorescence spectra. Despite the fact that the experiment we analyze in this work does not provide more insight into the sub-nanosecond dynamics of individual complexes than previous works, we nevertheless formulate the theory consistently in such a way that it enables the description of such processes. This is done in order to highlight the existence of a less studied type of processes on an intermediate time scale and to stress the need to search for experimental techniques which can cover the range of timescales from femtoseconds to nanoseconds in single molecular spectroscopy. We thus provide theoretical techniques to treat these future experiments. We have recently reported a progress towards measuring processes on the order of hundreds of femtoseconds in single light-harvesting complexes^29^.

The paper is organized as follows: we first discuss the Frenkel exciton model as a basis for the formulation of equations of motion for the populations of the excited states of chromophore aggregates with strong interchromophore couplings. We introduce equations of motion for excitonic populations valid over a broad (from ps to min) timescale range. We discuss their generality and the range of validity. It is argued that these equations provide an ideal means for the description of the SMS experiments. We apply our equatios to LHCII photosynthetic antenna complexes. All results are compared to the experiment. It is shown that our equations give correct fluorescence spectrum and peak statistics, i.e. appropriate steady-state population, under typical SMS experiment condition. Then we model the intensity traces, while the switching behaviour is included by incorporating one particular previously proposed NPQ mechanism, namely energy transfer to lutein Lut 1. The switching between on and off states is controlled by a 2-level model, where the switching causes a change of the Chl a612 - Lut 1 coupling. It is shown that using realistic parameters we are able to reproduce the experimental results. The details of our energy relaxation theory and of the stochastic model of switching are given in Supporting Information (SI).

## Results

### Frenkel-Exciton model

In the usual SMS experiments, time-resolved (on the times scale of 10 ms to s) fluorescence of the studied molecules is observed. If the excited state life time of the studied chromophores is sufficiently long (nanoseconds in the case of chlorophylls studied here), the expected fluorescence spectra can be calculated from the steady state, quasi-equilibriated populations of the excited electronic eigenstates of the molecular system, assuming canonical equilibrium. Depending on the strengths of the chromophore-chromophore resonance interaction and the system-bath couplings, the eigenstates can be approximated either by the excited eigenstates of the electronic subsystem or the excited state of the individual chromophores forming the aggregate. In the present paper, we treat both cases within one formalism provided by the framework of the Frenkel exciton model.

The Frenkel exciton model provides an excellent tool for the treatment of pigment-protein complexes on femto- and pico-second time scales^4^. The basic notion of the Frenkel exciton model is the one of localized excited states of the chromophores. These states have negligible wavefunction overlap with neighboring excited states, and they can therefore be assumed orthogonal to each other, forming a suitable basis for the aggregate Hilbert space. In the treatment of linear absorption and fluorescence experiments, only singly excited collective states of the molecular aggregate have to be included. The localized excited states can thus be denoted as 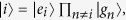 where |*g*_*i*_〉 and |*e*_*i*_〉 are the electronic ground and excited states of the chromophore denoted by index *i*, respectively. The states |*i*〉 form a complete Hilbert subspace for the case that exactly one molecule of the aggregate is excited. The molecular system Hamiltonian, *H*_*S*_, is however rarely diagonal in the basis of the states |*i*〉 and resonance couplings *J*_*ij*_ between excited electronic states |*i*〉 and |*j*〉 occur in all situations interesting for light-harvesting. In the absence of a protein environment, the light would resonantly excite eigenstates of the system Hamiltonian. In all realistic cases, the interaction of the system with its environment co-determines the excitation frequencies, and prescribes thus a “preferred basis” of electronic states in which it is the most advantageous to formulate the energy transfer theory.

In the case of the resonance interaction *J*_*ij*_ exceeding the typical reorganization energy *λ* of the protein bath of the chromophores *i* and *j*, (*J*_*ij*_ > *λ*), we include *J*_*ij*_ explicitly into the system Hamiltonian. The Hamiltonian is diagonalized to obtain electronic eigenstates, and the effect of the protein bath is included via perturbation theory yielding a Redfield type relaxation tensor. In the opposite case (*J*_*ij*_ < *λ*) we neglect the resonance coupling in the system Hamiltonian, and its effects are included perturbatively yielding Förster type energy transfer rates between localized excited states^4^,^30^,^31^.

### Excited State Dynamics across Time-Scales

Let us first focus on deriving a closed set of equations for the excited state populations. Extensive work was done in the last years on developing methods to accurately describe the system dynamics following an ultrafast excitation by external light. The traditional Redfield and Förster approaches were superseded by more accurate (exact in some cases) methods, such as HEOM^32^,^33^,^34^, TEDOPA^35^ and other methods^36^,^37^,^38^,^39^,^40^. These methods bring unprecedented accuracy at an increased numerical cost. It seems, however, that for analyzing many of the recent experiments, it is still possible to rely on the traditional tools, as they capture the physics of the problem (and often even the quantitative aspects of the problem) very well^34^.

Commonly, equations of motion for some relevant degrees of freedom (DOF), electronic states in our case, are derived by reducing the Liouville - von Neumann equation for the total density matrix to an equation for the so-called reduced density matrix (RDM). These equations describe time evolution of a molecular system for a fixed average configuration of the protein environment (assuming fast fluctuations around this fixed average configuration), and they are therefore suitable for the description of ultrafast laser experiments. However, on the timescale relevant in SMS (up to tens of seconds), usage of these equations is actually not appropriate. First of all, some transient effect at the short times affect even the long time properties of the system and the steady state population dynamics, and second, slow changes in the protein environment can entirely change the energy landscape including the case that one has to change the theoretical limit (localized states, delocalized states) in which one works. The latter case is especially difficult to treat and requires to go beyond the traditional master equation approaches which we apply here. In this work we therefore concentrate on the extension of the validity of the master equation approach towards long times under the assumption that the dependence of the Hamiltonian of the system on time is negligible within one absorption emission time scale (nanoseconds). As for the transient effects at short time, when dealing with fast dephasing of optical coherences, short time transient effects are responsible for the absorption lineshape. This aspect of the transient effects will be taken into account in full.

Spontaneous emission of photons by chlorophylls occurs with a nanosecond life-time. Another class of transient effects, namely dynamic electronic coherence due to excitation by light, is therefore also unimportant and its treatment can be avoided. We therefore derive approximative equations for the population dynamics only, with the validity in the range from picoseconds to tens of seconds. The full derivation including all the approximative steps can be found in section IV of the SI. Here we present a brief, intuitive version outlining the basic concepts.

Our Hamiltonian consists of the system, bath and system-bath interaction terms, and we formally assume some total density matrix *W*(*t*) which follows the Liouville–von Neumann equation. There are several methods how to arrive to a master equation (in a convolution-less form) for the RDM *ρ*(*t*) = *Tr*_*B*_*W*(*t*)^31^,^41^. A general master equations for the RDM treatment of photo-induced dynamics which originates from a perturbative treatment of the system-bath interaction reads:


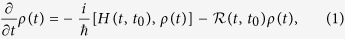


where


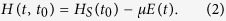


The total Hamiltonian *H* consists of the bath-renormalized system Hamiltonian *H*_*S*_ (*t*_0_) and the system-light interaction, which is (in the dipole approximation) given by the dipole moment operator *μ* and the electric field strength *E* (*t*). (When considering light polarization, *μ* represents the projection of the transition dipole moment vector on the polarization vector **e** of the field, *μ* = *μ* · **e**). The bath is completely eliminated in the reduced description, and its effects are represented by the relaxation tensor 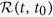. For the description of optical excitation, we can assume that the system is initially in the electronic ground state and the bath is in equilibrium with respect to this single state. In this case it is possible to eliminate exactly the so-called initial correlation term which should formally be present in Eq. (1). It is important to note that in Eq. (1), the relaxation tensor 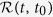 explicitly depends on some initial time *t*_0_, in which the initial condition for the propagation is set, or more precisely, the relaxation tensor depends on the quantity *t* − *t*_0_. An exact relaxation tensor would also depend on *E*(*t*). In a standard derivation of Eq. (1), the dependence of 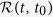 on the external field is neglected. Thus the reorganization of the bath, which is reflected in the time dependence of the relaxation tensor, starts formally at the time *t* = *t*_0_. In general, the choice of *t*_0_ is arbitrary, and we should make it such that the temporal profile of the excitation field is non-zero for *t* > *t*_0_ only. However, in most of the practical theoretical approaches, the evolution of the bath due to excitation of the system is reflected in the relaxation tensor 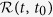 by the bath correlation function, which decays rather quickly on a time scale given by the so-called bath correlation time *τ*_*c*_. By choosing *t*_0_ such that *t* − *t*_0_ > *τ*_*c*_ for times *t* for which |*E*(*t*)| > 0, we could always make the relaxation tensor constant during the action of the pulse. This is obviously an artifact of the approximations used. For a smooth envelope of the external field changing on the same or slower timescale than *τ*_*c*_, there is no good choice of *t*_0_. The relaxation tensor always becomes constant before the action of the excitation pulse is over. Luckily, for weak external field excitation we do not need to account for the *E*(*t*) beyond low orders of perturbation theory. In this regime, there is a natural choice of time *t*_0_ which enables us to correctly account for the transient time dependence of the relaxation tensor even for a steady state externally driven by steady state light. It is important to note, that the relaxation tensor 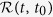 is completely abstract up to now. It can represent some exact relaxation superoperator, or a result of perturbation theory with respect to some parameters, such as the Redfield or Förster tensors.

Let us now formulate the equations of motion for the excited state evolution in the linear regime of the system’s interaction with the electric field. The validity of the linear regime has been discussed e.g. in refs 42, 43, 44, 45, and it is the same as the validity of the third order response theory for non-linear laser spectroscopy. We will also use the secular approximation (equations of motion for populations *ρ*_*ii*_ and coherences *ρ*_*ij*_, *i* ≠ *j* are assumed independent), although this we do only to simplify the resulting equations. Secular approximation could be avoided if one so wishes, at the cost of treating the full RDM. We will base our treatment here on Eq. (1), which is already a reduced equation of motion. Rigorous treatment which starts with the full density matrix *W*(*t*) is presented in Section IV of the SI, where we also discuss approximations involved in disposal of aditional terms which arise in the rigorous procedure. Keeping thus only terms of the first order of explicit appearance of the field *E*(*t*) in Eq. (1) we get the following set of coupled equations:


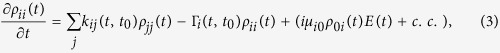



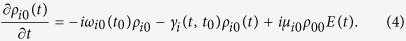


They have a form of ordinary linear differential equations with source terms. We write explicitly the elements of the RDM of Eq. (1) in which we keep the double time dependence of the Hamiltonian and the relaxation tensor. Here, the 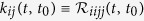 are transfer rates between populations (from *j* to *i*), 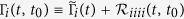 is the population relaxation rate of the state *i*, including the rate of radiative and nonradiative depopulation 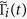, and 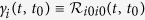 is the optical coherence dephasing rate. The time dependence of *γ*_*i*_(*t*, *t*_0_) is responsible for the absorption lineshape (see e.g. ref. 4) and the loss of this time dependence for long pulses would limit our treatment to Lorentz line shapes. Fortunately, there is a better way how to treat the excitation of optical coherences. Starting from the system ground state, the first interaction with the field creates an optical coherence according to Eq. (4). The second interaction with the field then creates the excited state population according to Eq. (3). The optical coherence therefore acts as a source term for the populations. The equation of motion for the optical coherences, Eq. (4), can be solved by introducing interaction picture using the evolution operator element 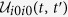, and integrating Eq. (4) in the interaction picture. By returning back to the Schrödinger picture, we obtain the actual field-induced and field driven optical coherence element in the form:


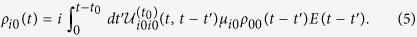


As expected, the optical coherence is proportional to the electric field *E*(*t*). The evolution operator element reads as


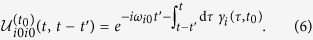


The element 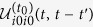 corresponds to an evolution of an optical coherence which was excited at time *t* − *t*′ by a Dirac delta pulse, *E*(*t*) ≈ *δ*(*t*). The time *t* − *t*′, not *t*_0_, is therefore a correct physical start of the bath reorganization. We fix the wrong start of the bath reorganization by correcting Eq. (6) by setting *t*_0_ = *t* − *t*′. It is important to note that such a correction of the behaviour is not possible directly in Eq. (4), and therefore Eq. (5) with *t*_0_ = *t* − *t*′ is actually not a solution of Eq. (4). In solving Eq. (4) numerically, the dephasing rate *γ*_*i*_(*t*, *t*_0_) would quickly become constant for *t* − *t*_0_ > *τ*_*c*_ and the transient effects for *t* − *t*_0_ ≈ 0 would be lost completely. However, in Eq. (5) these transient effects can be properly taken into account. The discrepancy between Eq. (4) and Eq. (5) with *t*_0_ = *t* − *t*′ is due to a different treatment of the bath. In a response function approach, of which the linearization of the full equations of motion with respect to the field is a variant, it is in general possible to account for the bath in a more consistent way than in master equations. We discuss this issue in more detail in Section IV.C of the SI. In ordinary master equations for RDM, the bath is correctly described at *t*_0_, and the description of its subsequent evolution after *t*_0_ is extremely limited. An example of such limitation is discussed e.g. in ref. 46.

In most biological energy transferring systems, the pure dephasing is much faster (hundreds of fs) than the population relaxation (units and tens of ps). It is therefore reasonable to set the upper limit of the integration in Eq. (5) to infinity by sending *t*_0_ → −∞. We can expect that the simultaneous action of pure dephasing and external driving by a field with a slowly varying envelope creates a steady state optical coherence. The dependence on *t*_0_ in the upper limit of the integral describes a transient evolution of the optical coherence after switching on the interaction with the field. Now that the short time time-dependent nature of the dephasing rates is taken into account correctly by properly placing the start of the bath reorganization to the time *t* − *t*′, we can set *t*_0_ → −∞ and write:


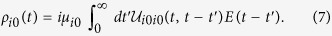


Here we defined 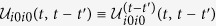. It is important to note that now *t* is a global time which can run through the whole minutes long SMS experiment. The evolution operator changes on an ultrafast time scale, but this timescale is scanned in the integration over the variable *t*′. The properties of the Hamiltonian *H*_*S*_ (*t*) change on a very slow time scale (with respect to optical dephasing), and so does the evolution operator element 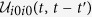. Unlike the Eq. (4), which is valid for *t* − *t*_0_ small, Eq. (7) is valid for all times.

The purpose of deriving Eq. (7) was to insert it eventually into Eq. (3). Also in Eq. (3) we face the problem of transient time dependence of the rates. However, because populations change much more slowly, these effects are not as important as in the case of coherences. They can be, however, treated rigorously, even including bath memory effects between evolutions by Eq. (3) and Eq. (4), as we have shown elsewhere^47^. A rigorous derivation of the equations of the excited state population as a second order equation in the external field is presented in the SI. Further on, we will assume the energy transfer and relaxation rates not to depend on the difference *t* − *t*_0_, although they may depend weakly on the time *t*, i.e. *k*_*ij*_(*t*, *t*_0_) = *k*_*ij*_(*t*) and Γ_*i*_(*t*, *t*_0_) = Γ_*i*_(*t*). Now, we are ready to insert Eq. (7) for optical coherences into the equation for populations, Eq. (3). We obtain


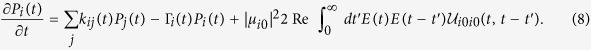


Here, we defined *P*_*i*_(*t*) = *ρ*_*ii*_(*t*), and we used the fact that *μ*_*i*0_ and even 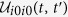 are ordinary c-numbers. Eq. (8) is the secular form of Eq. (74) from Section IV.F of the SI.

In Eq. (8), the populations are driven by a second-order field term. We have treated the field classically so far. If we did that quantum mechanically, we would now have to trace over the field DOF in order to obtain reduced equations of motion for the electronic state populations only. The term *E*(*t*)*E*(*t* − *t*′) would thus be replaced by 〈*E*(*t*)*E*(*t* − *t*′)〉 which can be interpreted as a quantum mechanical expectation value. The latter expression has the form of two-time correlation function of the electric fields of the light and its Fourier transform is the power spectrum of the light^42^,^44^,


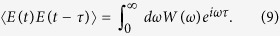


Now, inserting Eq. (9) into Eq. (8), switching the order of integrations, and using the definition of absorption lineshape of the i-th excitonic transitions (see e.g.^48^)


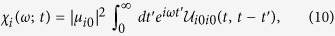


we arrive at


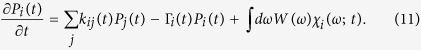


Our effort has yielded a closed set of equations for excitonic populations only. The population changes are given by the transfer rates between electronic levels, population quenching and a source terms expressed as an overlap of the excitonic spectra with the light spectrum. All quantities are in principle dependent on time, most importantly the excitonic absorption spectrum and all rates can weakly depend on time to simulate slow changes of the protein and chromophore configurations. Also the light spectrum can be considered time-dependent by generalizing the Wiener-Khintchine theorem for the instaneous power spectrum *W*(*ω*, *t*)^42^,^49^. The changes can be faster than the time resolution of the SMS experiment, but they have to be slower than the dephasing.

It should be noted that equations of the type of Eq. (11) in various forms and often times without source terms are frequently used to describe exciton dynamics (see e.g.^50^,^51^). The value of our description lies in presenting a rigorous route from total density matrix *W*(*t*) (in SI) to Eq. (11) and discussing all approximations involved. By this we achieve two things: First, the equations (11) include quantities such as the excitonic absorption spectrum with proper lineshape, light spectrum and population dynamics. These can be directly calculated on the quantum-mechanical level and subsequently used as an input. We do this in the following sections. And second, by careful consideration of all approximations involved in their derivation, we can infer the range of validity of our final equations. It is for these two reasons we can conclude that Eq. (11) is well-suited for description of SMS experiments.

### Spectroscopy of LHCII Complex

#### Excitonic Model for Bulk and Single Molecule Spectra

According to crystalographic studies, LHCII complex consists of three monomeric units, each containing 14 chlorophylls and four carotenoids: two luteins, neoxanthin and a carotenoid of the xanthophyll cycle^20^. The experiments described in this section were performed on monomeric LHCII complexes. In accord with the experiment, we focus on one such a monomeric unit in our model. We treat the LHCII monomer as a strongly coupled systems of chromophores, weakly coupled to the bath and weakly interacting with light. Because only absorption and fluorescence are measured, we do not attempt to fit the site energies in our simulations from scratch, as the fitting of this limited set of experiments would not be unique. Instead, we take the pigment transition energies from ref. 22, where both LHCII trimers and monomers where treated. The coupling energies between the pigments were calculated in the dipole-dipole approximation, and the dipole orientations were taken from the crystal structure using an effective dipole strengths of 3.4 D for Chl b and 4.0 D for Chl a. The bath is described by means of a spectral density obtained by fluorescence line narrowing experiment (FLN) (see refs 21,52). Excitonic absorption and fluorescence lineshapes are calculated by means of the second order cumulant expansion (see ref. 48), and the population transfer rates are calculated by the multilevel Redfield theory (see ref. 30). For comparison, we also calculated the rates by Modified Redfield theory, ref. 21, and we concluded that the results remain essentially the same. The population relaxation rates of chlorophylls due to fluorescence were taken to be 3 ns in accord with the experiment^53^. The equations of motion, Eq. (11), allow us to use light with any spectral composition. In the experiment described in this paper, we use spectrally narrow (laser) illumination at 630 nm. For details on the calculations see SI.

**Figure 1:**
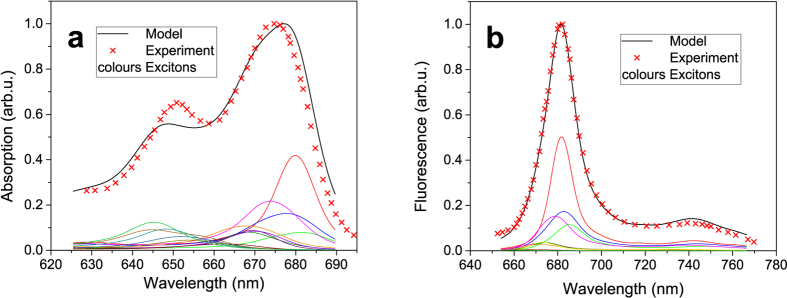
Bulk (**a**) Absorption and (**b**) Fluorescence spectrum of the Qy band of LHCII monomers. The points are experimental values taken from ref. 24, the lines are calculated by our exciton model. The coloured lines are individual excitonic contributions, the black line is the overal spectrum.

In Fig. 1a,b we present the calculated bulk absorption and fluorescence spectra of LHCII monomers at 5 °C compared to experimental values taken from^24^. We note here that the bulk spectra of LHCII in monomeric and trimeric form are practically identical, see Fig. S1 in SI for comparison. In calculations, the spectra were averaged over a Gaussian disorder of site energies with full width at half maximum (FWHM) of 110 cm^−1^. Although the blue Chl b shoulder is not perfectly reproduced, the agreement between measured and theoretical absorption is good in the region of our excitation, and the fluorescence (FL) spectrum shows a good agreement in general. We therefore conclude that our excitonic model captures correctly the features of the studied system that are the most relevant in the present study.

The FL spectrum in Fig. 1b is dominated by the lowest four excitons, which are the most populated ones. These excitons are formed by strongly coupled pigments Chl 610-611-612, Chl 602-603 and Chl 613-14 (see ref. 20 for nomenclature). This is in agreement with previous modeling results for the trimeric LHCII^22^.

**Figure 2:**
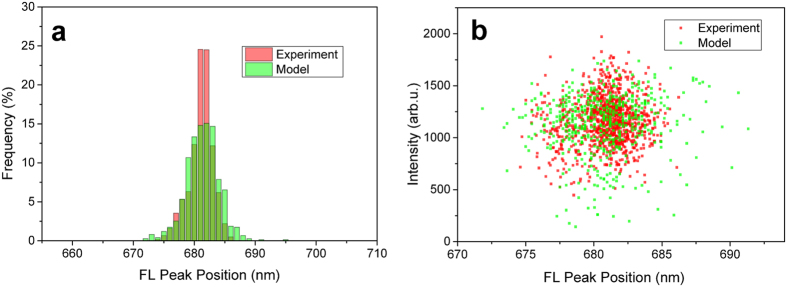
Experimental (red) and calculated (green) fluorescence peak distribution. (**a**) Peak position histogram, (**b**) FL peak position and intensity plot. Theoretical points are calculated as individual realizations of energetic disorder.

The calculated bulk spectra seem to be in a good agreement with the experiment. Our model also reproduces successfully the statistics from the SMS experiments. In Fig. 2a we present the FL spectral peak distribution compared to the experiment. We can see that the calculated distribution is a little broader than the experimental one, but the agreement is again reasonably good. The larger distribution width of the calculated spectra can be explained by a relatively long integration time in the experiment (1 s), during which the system samples several individual realizations of the disorder. In extreme cases the measured values get averaged towards the mean value. As a result the measured distribution is narrower. From our comparisons between experiments and calculations we conclude that the exciton model with Gaussian disorder not only reproduces the averaged absorption spectra and equilibriated populations of excitons (resulting in characteristic FL spectra), but also the individual realizations provided by this model are in a good agreement with the experiment.

The next feature of the LHCII single molecular spectra that we need to address is the significant amount of blinking, i.e. reversible switching to the off state. Since the measured dwell times in the off state are in the range of seconds, which is significantly longer than the lifetime of any long-living species such as triplet states^54^, the off states must correspond to states with efficient excitation energy dissipation. Correspondingly, our model has to be extended by including some fluorescence quenching mechanism.

#### Lutein Lut 1 as a Fluorescence Quencher

One of the possible mechanism of FL quenching in LHCII, proposed by Ruban and coworkers^7^,^55^, is an excitation energy transfer from the lowest chl a exciton states to a lutein molecule, Lut 1 (lut620), see ref. 20 for nomenclature). The Lut 1 molecule resides in the vicinity of the so-called terminal emitter group of chlorophylls, composed of Chls a610, 611 and 612, and it is supposed to be coupled mainly to Chl a612^7^,^55^. The S1 state of the Lut molecule is optically forbidden, and it has a short (10 ps) lifetime due to a decay through a non-radiative channel^56^. The transition energy from the S0 → S1 of Lut is in the vicinity of the transition to the Chl *Q*_*Y*_ state. Due to its short excited state life-time, Lut could in principle act as an excitation (and fluorescence) quencher. Let us test this mechanism within our model to see if it can account for the observed blinking. The important parameters of the lutein in context of our model are its S1 state site energy relative to its groundstate and the coupling to chlorophylls, in particular to Chl a612.

**Figure 3:**
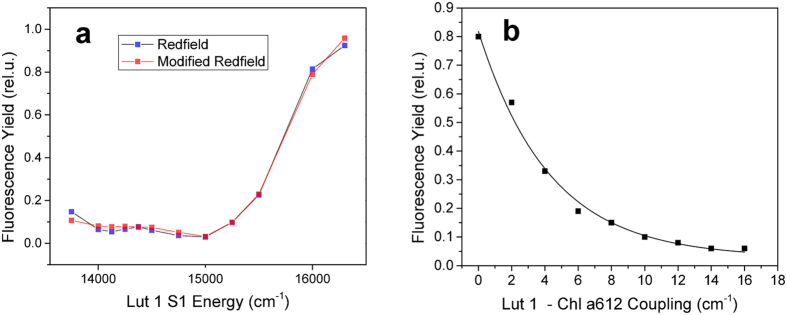
Role of Lut 1 parameters. Dependence of the fluorescence yield on (**a**) Lut 1 S1 energy and (**b**) Lut 1 - Chl a612 coupling. The energy dependence is calculated with Redfield (blue) and Modified Redfield (red) theory for comparison. The dependence on the coupling strength depicts calculated points (Redfield theory) fitted with exponential dependence. Already a realistically small coupling around 12 cm^−1^ leads to significant FL quenching.

In Fig. 3a we present the dependence of the relative FL quantum yield on the Lut energy for fixed value of the Lut-Chl coupling of 14 cm^−1^. The energy dependence agrees well with the one obtained by Ruban^55^. The quenching is only efficient when the Lut energy is below one of the red chlorophylls (around 15100 cm^−1^) and the plateau enables Lut to act as an efficient quencher even in disordered systems.

In Fig. 3b we show the dependency of the FL quantum yield on the Lut-Chl coupling for fixed Lut energy 14500 cm^−1^. Due to large reorganization energy, 14500 cm^−1^ corresponds to the zero-phonon line at 13900 cm^−1^ and thus agrees with experimental observations^56^. From the coupling dependence of fluorescence in Fig. 3b we can conclude that weak coupling is sufficient for Lut 1 to act as a fluorescence quencher. Very importantly, even small changes in the Lut-Chl coupling can result in a big difference in the fluorescence intensity. Based on this analysis we decided to use lutein S1 site energy of 14500 cm^−1^ in our simulations of blinking. We define the quenched state by the value of 12 cm^−1^ for coupling of the Lut 1 to Chl 612 and the unquenched state by the zero coupling. Our model allows any type of time dependency of the Chl-Lut coupling to be used, and it could in principle accommodate input from structure based MD studies and quantum chemical treatment of the (Dexter type) coupling of the Chl *Q*_*Y*_ and the Lut S1 states. However, a much rougher phenomenological model of the Chl-Lut coupling changes enables a better discussion of the feasibility of the proposed quenching mechanism than the parameter free *ab initio* calculations suffering from uncertainties in the structural information. Next we proceed to model the blinking statistics.

#### Model of the On-Off State Switching

As mentioned in the Introduction, the blinking statistics alone can be well described by a two-level model proposed by Valkunas *et al.* in ref. 57. By random fluctuations, the protein samples its potential energy surface (PES) performing thus a random walk (RW). The model of ref. 57 assumes that there are two stable conformations of the protein corresponding to two minima of the protein PES. These are approximated by two harmonic potentials. The protein undergoes a RW in this potential, and at every step it has a certain probability to switch from its current PES to the other PES. In our treatment we use a discrete RW description, which enables us to follow individual trajectories of the proteins. For the details of the approach taken in this study and the differences from the original model by Valkunas *et al.*, see SI and refs 57,58.

**Figure 4:**
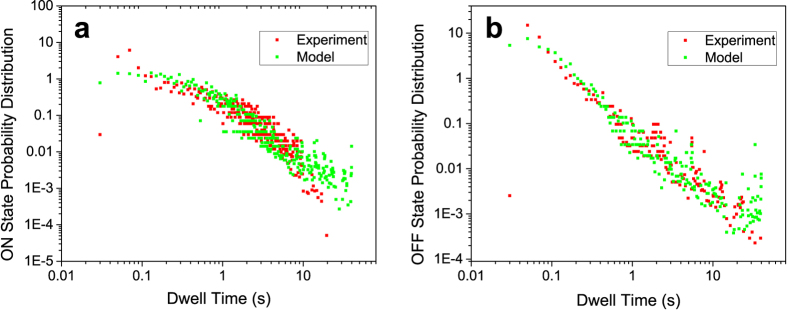
Experimental (red) and calculated (green) probability distribution of dwell times in the (**a**) ON and (**b**) OFF state, logarithmic scale. While the OFF state distribution follows a power law, the ON state distribution has an exponential tail at longer times.

To connect the two PES model to our particular Lut quenching model, we assume that the change of protein conformation somehow changes the Lut-Chl coupling. The Lut S1 state does not have a dipole moment, and the resonance coupling similar to those between allowed states does not occur here. The two different protein conformations responsible for the quenched/unquenched states would then result in two slightly different orientations/positions of the pigments, leading to different strengths of the coupling. This mechanism is in accord with recent quantum-chemical study by Duffy *et al.*^59^, where small configurational changes were found to lead to substantial changes in chl-car couplings. The switching between the PES is controlled by the RW model with diffusion parameters adjusted to fit the experimental dwell time distributions. The comparison between the calculated and experimentally determined dwell-times is presented in Fig. 4a,b, for the on and off times, respectively. The agreement is again fairly good letting us believe that our phenomenological model captures the most important features of the protein dynamics affecting the blinking behaviour.

#### Intensity traces

**Figure 5:**
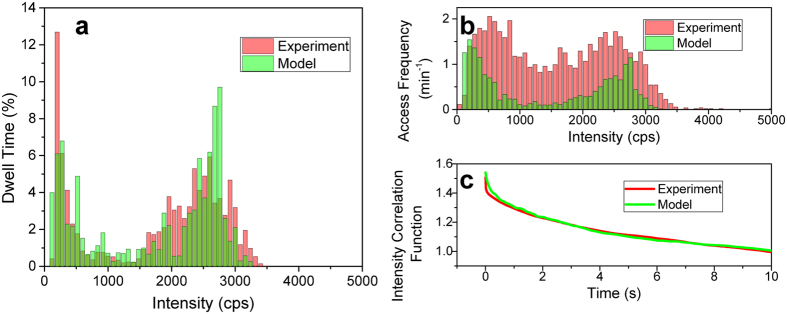
Experimental (red) and calcuated (green) intensity blinking statistics. (**a**) The percentage of time the complexes dwelled on respective intensity levels. The two-state structure of the low-intensity OFF states and higher intensity ON states is apparent. (**b**) How often per minute the complexes accessed the respective intensity levels. The experimental frequency is higher due to switching within the ON/OFF states. (**c**) The intensity correlation function.

Finally, we can connect the two models described above and simulate the blinking behaviour. To this end we continuously model the fluorescence of the LHCII complexes, and output the intensity (and spectrum) every 10 ms corresponding to the experimental integration bins. Simultaneously, we let the protein do the RW on its PES, and we adjust the Chl/Lut coupling, when the protein switches between PES. To obtain more realistic traces, either the site energies or couplings can be slightly varied after every jump, resulting in different energy levels. Such a procedure, however, does not lead to qualitatively different conclusions and it can be in principle omitted. In the calculations presented here, we used Gaussian disorder with the FWHM of 0.3 cm^−1^ for couplings and 1 cm^−1^ for energies. For every realization of the disorder a 60 s trace is modelled. This is repeated for 200 realizations of the disorder, reflecting the experimental conditions. The resulting statistics are presented in Fig. 5.

Total dwell times in Fig. 5a represent the overall amount of time spent in a given intensity level. From this we can see the two-level character of the blinking and simultaneously also the presence of some intermediate levels, which result from particular disorder realizations. The dwell times are similar for the on and off states. Figure 5b shows how often are the intensity levels visited per fixed amount of time. The modelled access frequency distribution is naturally very symmetrical, a direct result of the fact that in the model the complex switches only between the on and off state. The number of on/off states visited per minute therefore has to be the same. The experimentally analyzed intensity traces contain also jumps between levels within the on/off states in an amount which can be, to some extent, modified by adjusting the sensitivity of the level analysis. The presence of these intra-state jumps results in higher switching frequency and wider distribution in intensities, causing a moderate discrepancy between experiment and simulation. In order to include this kind of switching into the model, dynamic sampling of the disorder would have to be incorporated. Work in this direction will be presented elsewhere.

For the reasons stated above, the level access frequency distribution is not well suited for comparison of the model with the experiment. A more appropriate measure of the blinking would be the intensity-intensity correlation function defined as *h*^(2)^ (*τ*) = 〈*I*(*τ*)*I*(0)〉/〈*I*(0)〉^2^. This quantity is well-known from single-molecule measurements, where it is often used to characterize the blinking behaviour^60^. In Fig. 5c we present *h*^(2)^ (*τ*) obtained from 50 measured long enough traces, compared with the model. We can see that the agreement between experiment and theory is good, indicating that our model gives reasonable switching between the intensity levels. The shape of the correlation function is given by the dwell time statistics, see also Fig. 4. The initial fast drop implies the abundance of short blinking events. This results from the mechanism of the protein switching between its potential surfaces, where the short succesive blinking events are caused by the dynamics in the vicinity of the PES intersection.

## Discussion

We have derived approximate equations of motion for populations evolution on the timescale of the SMS experiment. We have shown that the weak illumination regime, in which the SMS experiment is performed, allows for an effective source term description of the light-matter interaction in which short time transient effects arising in the photoinduced evolution of molecular systems can be consistently accommodated side by side with the slow evolution of the protein bath observed in SMS experiments. We demonstrated the validity and scope of application of our equations by simulating our single molecule experiment on LHCII monomers. Based on the recent research in elucidating the NPQ mechanism in LHCII and the connection between fluorescence quenching and energy dissipation we implemented energy transfer to Lut 1 as a blinking mechanism. Based on our calculations we were able to confirm the findings of Duffy *et al.*^55^ and Chmeliov *et al.*^59^ that, within a reasonable range of parameters, Lut 1 can indeed act as an efficient quencher. Since our model extends the previous treatment by including realistic excitation conditions and population transfer rates, it is remarkable, how similar our fluorescence quenching dependence on the Lut 1 energy is to the one in ref. 55. Moreover, we were able to confirm that Lut 1 acts as an efficient quencher also under AM1.5 illumination (data not shown since the dependence is very similar). At the same time we can see that the amount of quenching is very sensitive to the change of coupling of the Lut to the chlorophylls. Since the coupling itself is very sensitive to the distance and orientation between the pigments, it provides a possible link to the protein conformation change working as a switching mechanism as proposed in refs 57,59. Indeed, when using the 2-level switching model to control the change of coupling, we are able to reproduce the experimentally obtained blinking statistics. Although far from being exclusive in any way, our argument strongly supports the notion of the protein acting as a conformational switch regulating the amount of quenching in the system.

The agreement between the theory and experiment also serves as a good demonstration of the scope of our equations. They provide a description for controlling the energy transfer in the system by modulating the parameters of the excitonic model. We are aware of the remaining phenomenological nature of our connection of the 2-level switching model with the excitonic model. Further improvements in the direction of introducing more parameters with a particular physical meaning, for example the relation to the actual PES shape, are needed and will be subject to further study. Also recent experimental observations indicate the presence of more relevant timescales in the intensity traces suggesting the inadequacy of a 2-level model with one reaction coordinate. Finally, although some connection between the fluorescence blinking and NPQ was already shown by Krüger *et al.*^26^, their exact relation is yet to be elucidated. The equations derived here are a suitable tool for these future investigations.

## Methods

### Sample preparation

Trimeric LHCII complexes of spinach were isolated from freshly prepared thylakoid membranes as described earlier^61^. Monomeric complexes were obtained by incubating LHCII trimers with 1% (w/v) octyl glucoside and 10 *μ*g/mL phospholipase A2 (Sigma)^62^. Subsequent fast protein liquid chromatography (FPLC) ensured a homogeneous sample preparation. The ensemble fluorescence absorption and emission spectra were measured on a Lambda40 spectro-photometer (Perkin-Elmer) and a FluoroLog Tau-3 (Jobin Yvon), respectively. For SMS experiments, the sample was diluted down to a concentration of ~10 pM in a measuring buffer (20 mM Hepes, pH 8 and 0.03% (w/v) n-Dodecyl *β*-D-maltoside) and then immobilized on a PLL (poly-L-Lysine, Sigma) coated cover glass. Addition of an oxygen scavenging mix of 750 *μ*g/ml Glucose Oxidase, 100 *μ*g/ml Catalase and 7.5 mg/ml Glucose (all Sigma) to the closed sample chamber inhibited the formation of highly reactive singlet oxygen and improved the photostability of complexes.

### Single-molecule detection

A confocal microscope was used to measure the fluorescence of single complexes at 5 °*C* as described previously^24^. The sample was excited at 630 nm utilizing a Ti:sapphire laser (Coherent MIRA 900F) with a pulse width of 200 fs and a repetition rate of 76 MHz coupled to a tunable optical parametric oscillator (Coherent MIRA OPO). Near-circular polarized light was obtained by utilizing a Berek polarization compensator (5540 New Focus). A fluorescence beam splitter (70:30, Thorlabs) allowed us to simultaneously measure the fluorescence spectrum *via* a CCD camera (Spec10:100BR, Roper Scientific) with an integration time of one second and the wavelength integrated fluorescence intensity *via* an avalanche diode (SPCM-AQR 16, Perkin Elmer) with a binning time of 10 milliseconds. The fluorescence of one complex was analyzed for either one minute or until it photo-bleached and a set of 200 complexes served as the basis for statistical analysis. The fluorescence peak distribution was obtained by fitting of a skewed Gaussian to the fluorescence spectrum as shown in Kruger *et al.*^24^ and the blinking analysis was performed equivalently to the algorithm described elsewhere^28^.

### Dynamics simulation

The equations of motion, Eq. (11), have quasi constant coefficients, and they can therefore be written in the form


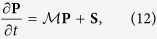


where 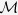 is a matrix of relaxation and population transfer rates, and **S** are the source terms. Eq. (12) can be solved analytically:


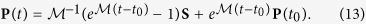


This expression enables us to find the populations at any time without the need to solve for all the previous times. If we aim at steady state, *t*_0_ can be send to −∞ and the populations then depend only parametrically on time *t*. The weak dependence of **S** and 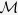 on *t* makes it possible to explain changes in the populations of the emitting states of a molecular system due to slow changes of the protein environment and the structure of the molecular system.

## Additional Information

**How to cite this article**: Malý, P. *et al.* Single Molecule Spectroscopy of Monomeric LHCII: Experiment and Theory. *Sci. Rep.* **6**, 26230; doi: 10.1038/srep26230 (2016).

## Supplementary Material

Supplementary Information
