# Supplementary material for: Single Molecule Spectroscopy of Monomeric LHCII: Experiment and Theory
Source: Sci Rep. 2016 May 18;6:26230. doi: 10.1038/srep26230 (PMC4870570; doi:10.1038/srep26230)
Supplement: Supplementary Information [file srep26230-s1.pdf]

Single Molecule Spectroscopy of Monomeric LHCII: Experiment and Theory
Supporting Information

Pavel Malý[1,2], Michael Gruber[2], Rienk van Grondelle[2] and Tomáš Mančal[1,2]

[1]*Institute of Physics, Faculty of Mathematics and Physics,*

Charles University in Prague, Ke Karlovu 5,

CZ-121 16 Prague 2, Czech Republic and

[2]*Department of Physics and Astronomy,*

Faculty of Sciences, Vrije Universiteit Amsterdam,

1081 HV Amsterdam, The Netherlands

I. EXCITON MODEL

The interaction of the pigments, i. e. the system, with the vibrations, i. e. its environment, is treated by second order perturbation theory. There the bath is completely described by its correlation function or, equivalently, in the frequency domain, by the spectral density of the bath vibrations. We use the spectral density from Ref. [1]. It is constructed from one overdamped oscillator representing the slow protein vibrations and 48 high-frequency intrapigment modes (for more context for the spectral density and excitonic model see Ref. [2]):

$$C''(\omega) = 2\lambda_0 \frac{\gamma_0 \omega}{\omega^2 + \gamma_0^2} + \sum_{i=1}^{48} 2S_j \omega_j \frac{\omega_j^2 \gamma_j \omega}{(\omega^2 - \omega_j^2)^2 + \omega^2 \gamma_j^2}. \tag{1}$$

The (temperature dependent) correlation function given by this spectral density is assumed to be uncorrelated between individual sites and differs only between Chl a and Chl b, while the difference is in the coupling strength $\nu_n = \nu_{a/b}$:

$$C_n(\omega) = \nu_n C(\omega) = \nu_n \left(1 + \coth\left(\frac{\hbar\omega}{2k_B T}\right)\right) C''(\omega). \tag{2}$$

The time-dependent correlation function is then obtained by Fourier transform of (2). We use the same values of the spectral density parameters as in Ref. [1]. The difference between the vertical, Franck-Condon transition of the pigments, which are called site energies in this text, and their 0-0 transitions is given by the reorganization energy due to the interaction with the bath:

$$\lambda = \frac{1}{\pi} \int_0^\infty \frac{C''(\omega)}{\omega}. \tag{3}$$

Because the pigments are strongly coupled, the preferred basis of calculations is the excitonic basis in which the system Hamiltonian is diagonal with eigenvalues, exciton energies, ω_{i0}. All quantities, including correlation functions $C(t)$, reorganization energies λ and transition dipole moments μ have to be transformed into the excitonic basis:

$$C_i(t) = \sum_n |c_i^n|^2 C_n(t), \tag{4}$$

$$\mu_{i0} = \sum_n c_i^n \mu_{n0}, \tag{5}$$

$$\lambda_i = \sum_n |c_i^n|^4 \nu_n \lambda. \tag{6}$$

The position of the zero phonon lines of the excitonic transitions is then

$$\omega_{i0}^{ZPL} = \omega_{i0} - \lambda_i. \tag{7}$$

The spectral lineshapes are calculated by 2nd order cumulant expansion employing the so-called lineshape functions:

$$g_{ii}(t) = \int_0^t d\tau \int_0^\tau d\tau' C_i(\tau'). \tag{8}$$

The lineshape function is conveniently expressed in terms of the spectral densities

$$g_{ii}(t) = \frac{1}{\pi} \int_0^\infty d\omega \frac{C_i''(\omega)}{\omega^2} \left[\coth\left(\frac{\hbar\omega}{2k_B T}\right) (1 - \cos(\omega t)) + i\left(\sin(\omega t) - \omega t\right) \right]. \tag{9}$$

The absorption spectrum is calculated as

$$abs(\omega) \propto \omega \sum_i \chi_i(\omega), \tag{10}$$

where the absorption lineshape is

$$\chi_i(\omega) = |\mu_{i0}|^2 \mathrm{Re} \int_0^\infty d\tau\, e^{-i(\omega-\omega_{i0})\tau - g_{ii}(\tau) - \frac{\Gamma_i}{2}\tau}. \tag{11}$$

Here Γ_i is the population relaxation rate from state i. The fluorescence is similarly given as

$$FL(\omega) \propto \omega^3 \sum_i P_i \tilde{\chi}_i(\omega), \tag{12}$$

where P_i is the steady-state population of state i and the fluorescence lineshape is

$$\tilde{\chi}_i(\omega) = |\mu_{i0}|^2 \mathrm{Re} \int_0^\infty d\tau\, e^{-i(\omega-\omega_{i0}+2\lambda_i)\tau - g_{ii}^*(\tau) - \frac{\Gamma_i}{2}\tau}. \tag{13}$$

The population transfer rates in the Redfield theory are obtained as

$$k_{ij} = \sum_n |c_i^n|^2 |c_j^n|^2 C_n(\omega_{i0}^{ZPL} - \omega_{j0}^{ZPL}). \tag{14}$$

The population relaxation rates of chlorophylls are a result of energy transfer and radiative and non-radiative decay, i.e. $\Gamma_i = \sum_j k_{ji} + \tilde{\Gamma}_i$, where $\tilde{\Gamma}_i = \sum_n |c_i^n|^2 \tilde{\Gamma}_n$, $\tilde{\Gamma}_n = 1/\tau_n$ is inverse lifetime τ_n of site n excited state, which is taken to be 3 ns for all n.

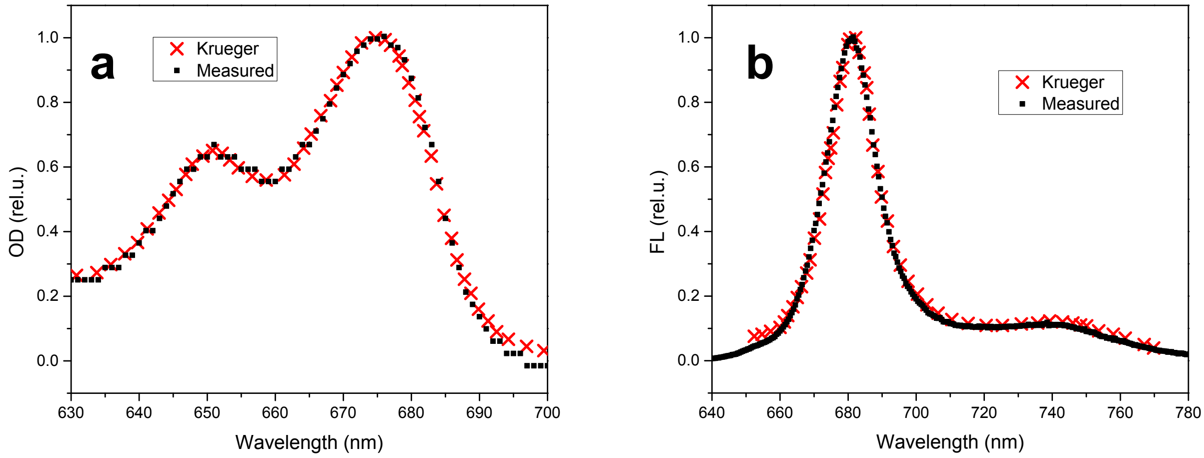

Figure 1: Bulk spectra measured and taken from Ref. [3]

II. BULK SPECTRA MEASUREMENT

To check for sample degradation a control bulk measurement of absorption and fluorescence was performed. In Fig. 1 the measured spectra are given together with the spectra of LHCII trimers taken from [3]. Their excellent agreement confirms absence of degradation in course of the experiment and also justifies the usage of the trimer spectra for the bulk spectra modeling in the main text.

III. RANDOM WALK MODEL

In the original description by Valkunas [4] and, in more detail, in [5], the protein diffusive motion was described as a continuous-time random walk (CTRW) on a two-dimensional potential energy surface. Here we somewhat simplify this description in the following way. The two generalized coordinates represent fast and slow degrees of freedom and thus, when following the slow dynamics, the fast fluctuations can be adiabatically eliminated. It can be shown that when we consider the potential dependence on the fast coordinate the same in both the on and off states for simplicity, i.e. setting $x_0 = 0, {}^{\lambda_2}/_{\lambda_1} = 1$ in [4], the fast coordinate can be completely removed. This leaves us with effectively one-dimensional problem.

After transforming into dimensionless coordinates $y\sqrt{\frac{\gamma_1}{k_B T}} \to y$, the parabolically approximated minima of the potential for the on (1) and off (2) state are

$$\frac{U_1}{k_B T} = \frac{1}{2} y^2,$$
$$\frac{U_2}{k_B T} = \frac{1}{2}\frac{\gamma_2}{\gamma_1}(y-y_0)^2 + \frac{U_0}{k_B T}. \quad (15)$$

Here $\gamma_{1,2}$ determine the steepness of the potential and the second minimum is shifted by y_0 along the slow coordinate and by U_0 along the potential energy.

The protein then performs random walk on the respective surface, with probabilities to tunnel to the other surface

$$\kappa_{1\to2}(y) = k_1 \; e^{-\alpha|U_1-U_2|/\hbar\omega_0} \min\{1, e^{(U_1-U_2)/k_B T}\},$$
$$\kappa_{2\to1}(y) = k_2 \; e^{-\alpha|U_1-U_2|/\hbar\omega_0} \min\{1, e^{(U_2-U_1)/k_B T}\}. \quad (16)$$

k_i is a rate of falling from the $i-th$ potential, the first exponential term reflects the energy gap law and the min term ensures detailed balance condition. The coefficient α can be treated as a constant and ω_0 is a characteristic frequency of the protein environment vibrations responsible for the tunneling.

The protein diffusion under this conditions can be described either by the CTRW or by a discrete random walk (RW). The former approach was employed by Valkunas et al.[4, 5]. However, we believe that for our purpose it is better to solve this problem as a discrete RW for two reasons. First, the coupled Smoluchovski equations for the CTRW on the two potentials can be decoupled only for conditional probabilities, i.e. assuming that the system was in the opposite state in the previous interval, and, in the same time, employing the same, equilibrium initial condition for each dwell time. When continuously modeling the trajectory of a single protein, we do not have to include the resetting after switching and also the conditioning will be inherent, as the system is observed being in a particular state. And second, when we want to simulate the individual intensity time traces, it is more natural to really follow the trajectories of the individual proteins on their PES.

If we want to follow the time trace of every single molecule, we should follow its particular trajectory. The blinking statistics will then be recovered by averaging over a large number of molecules, exactly as in the experiment. To the purpose of following trajectories of individual proteins, we need to describe its discrete RW (DRW) in the potential. We will

denote probability of going right (left) as p (q). In the symmetrical RW we have $p = q = \frac{1}{2}$. If the protein is at a coordinate y, in the next step it will move with probability p to $y+a$ and with probability q to $y-a$, where a is the length of the step. Inspired by classical approach by van Kampen [6], we augment the position dependent probabilities in the presence of the potential $U(y)$ as

$$\begin{aligned} p(y) &= \frac{1}{2} e^{-\frac{1}{k_B T}(U(y+a)-U(y))}, \\ q(y) &= \frac{1}{2} e^{-\frac{1}{k_B T}(U(y-a)-U(y))}. \end{aligned} \tag{17}$$

We note that defined probabilities defined in Eq. (17) reflect the detailed balance condition $\frac{p(y)}{q(y)} = e^{-\frac{1}{k_B T}(U(y+a)-U(y-a))}$. Considering a small step a, we can use Taylor expansion in y $\left(e^{-\frac{1}{k_B T}U(y\pm a)} \approx e^{-\frac{1}{k_B T}U(y)}\left(1 \mp \frac{1}{k_B T}\frac{dU}{dy}(y)a\right)\right)$, obtaining

$$p(y) - q(y) = -\frac{1}{k_B T}\frac{dU}{dy}(y)a. \tag{18}$$

Now, considering that $p(y) + q(y) = 1$, we get

$$p(y) = \frac{1}{2} - \frac{1}{2k_B T}\frac{dU(y)}{dy}a. \tag{19}$$

Using the potential form (15), we get for the probabilities

$$\begin{aligned} p_1(y) &= \frac{1}{2} - \frac{1}{2k_B T} y a_1, \\ p_2(y) &= \frac{1}{2} - \frac{1}{2k_B T}\frac{\gamma_2}{\gamma_1}(y - y_0)a_2. \end{aligned} \tag{20}$$

The length of the step a on respective surface can be related to the transformed diffusion coefficient $D_{1,2}$:

$$a_{1,2} = \sqrt{\frac{D_{1,2}\gamma_1}{k_B T}\Delta t}, \tag{21}$$

where Δt is the time duration of the step. During Δt the protein walks either to the left or right with the respective probability and with the probability $\kappa_{i\to j}(y)\Delta t$ switches to the other surface.

Similarly to [4] the position near the potential intersection can be chosen as an initial condition. However, as we do not include resetting after switching in our approach, this

parameter	Valkunas [4, pH 8]	This work
λ_2/λ_1	0.2	1.0
γ_2/γ_1	0.72	0.72
$x_0\sqrt{\lambda_1/k_BT}$	1.0	0.0
$y_0\sqrt{\gamma_1/k_BT}$	8.57	6.07
U_0/k_BT	1.5	1.5
k_1^{-1}	430 ms	330 ms
k_2^{-1}	4.8 ms	50 ms
$(D_1\gamma_1/k_BT)^{-1}$	3.8 s	66 s
$(D_2\gamma_1/k_BT)^{-1}$	1.4 s	10 s
$\hbar\omega_0/\alpha k_BT$	1.0	1.0

Table I: RW model parameters

determines only the starting point of each trajectory and is therefore not of significant importance. In Table I we present the parameters of our RW model compared to the ones used by Valkunas et al [4]. Considering the differences - LHCII monomers vs trimers, discrete vs continuous RW, no resetting vs resetting - the agreement is satisfactory.

IV. DERIVATION OF RDM EQUATIONS OF MOTION IN WEAK-FIELD LIMIT

We start with Liouville–von Neumann equation (LvNE) with a Hamiltonian

$$H = H_M - \mu E(t), \tag{22}$$

where

$$H_M = H_S + H_B + H_I \tag{23}$$

is the matter Hamiltonian including part, H_S, describing the relevant system of interest (electronic degrees of freedom (DOF)), thermodynamic bath H_B, in which the system is embedded and system–bath interaction term H_I. Interaction between the light and matter is described by semiclassical light-matter Hamiltonian in dipole approximation. We denote the full density matrix of the system by $W(t)$. The LvNE reads as

$$\frac{\partial}{\partial t}W(t) = -\frac{i}{\hbar}\left[H_M, W(t)\right]_- + \frac{i}{\hbar}\left[\mu, W(t)\right]_- E(t). \tag{24}$$

Our aim is to discuss approximations necessary to make a transition towards equations of motion for the so-called reduced density matrix (RDM)

$$\rho(t) = \mathrm{tr}_{\mathrm{bath}} \{W(t)\}, \tag{25}$$

and ultimately to the exciton populations

$$P_a(t) = \rho_{aa}(t) = \langle a|\rho(t)|a\rangle, \tag{26}$$

where $|a\rangle$ are eigenstates of the Hamiltonian H_S.

A. Expansion in Orders of Electric Field

In order to make approximations only with respect to the system-bath interaction Hamiltonian, we first switch to the interaction picture with respect to the field free evolution of the system. We define

$$W^{(I)}(t) = U(t_0,t)W(t)U(t,t_0), \tag{27}$$

which leads to

$$\frac{\partial}{\partial t}W^{(I)}(t) = \frac{i}{\hbar}\left[U(t_0,t)\mu U(t,t_0), W^{(I)}(t)\right]_- E(t). \tag{28}$$

Here, we used the field free evolution operator

$$U(t,t_0) = \exp\left\{-\frac{i}{\hbar}H_M(t-t_0)\right\}, \tag{29}$$

which propagates the density matrix from its initial condition at time t_0 to time t. Eq. (28) will be formally integrated, and we return to Schrödinger picture. This yields

$$W(t) = U(t,t_0)W(t_0)U(t_0,t) + \frac{i}{\hbar}\int\limits_{t_0}^{t} \mathrm{d}\tau\; U(t,\tau)\left[\mu, W(\tau)\right]_- U(\tau,t)E(\tau). \tag{30}$$

We obtained a recursive formula for the total density matrix $W(t)$. By iterative reinsertion of Eq. (30) into itself, we obtain a series of terms in orders of the electric field $E(t)$. We get

$$W(t) = \sum_n W^{(n)}(t), \tag{31}$$

where

$$W^{(0)}(t) = U(t,t_0)W(t_0)U(t_0,t), \tag{32}$$

and

$$W^{(n)}(t) = \frac{i}{\hbar} \int_{t_0}^{t} \mathrm{d}\tau\; U(t,\tau) \left[\mu W^{(n-1)}(\tau)\right]_- U(\tau,t) E(\tau), \tag{33}$$

for $n > 0$.

For any order of perturbation, we can write a differential equation instead of the integral expressions, Eq. (33). By differentiating Eq. (33) we get

$$\frac{\partial}{\partial t} W^{(n)}(t) = -\frac{i}{\hbar}[H_M, W^{(n)}(t)] + \frac{i}{\hbar}\left[\mu, W^{(n-1)}(t)\right]_- E(t). \tag{34}$$

The second term on the right hand side of Eq. (34) is a source term which is a known function of time, if the electric field is known and the lower order density matrix is known.

B. Equations of Motion for the Reduced Density Matrix with Source Term

So far, one could equivalently describe the density matrix $W^{(n)}(t)$ by both Eqs. (33) and (34). However, performing a straightforward trace over the bath DOF (like in Eq. (25)) is only possible in Eq. (33) which presents the solution of Eq. (34). Tracing over Eq. (34) does not lead directly to a closed set of equations for the RDM $\rho(t)$. Rather, one has to derive a master equation which includes terms describing phenomena emerging due to the reduction from a full set of DOF to a defined subset of the system's DOF.

We will formally separate the matter Hamiltonian H_M into the relevant electronic DOF described by Hamiltonian H_S, bath DOF described by the Hamiltonian H_B and some interaction Hamiltonian H_I. We define

$$H_0 = H_S + H_B. \tag{35}$$

We switch into interaction picture with respect to the interaction free part of the matter Hamiltonian by defining

$$\bar{W}^{(n)}(t) = U_0(t_0,t) W^{(n)}(t) U_0(t,t_0), \tag{36}$$

were

$$U_0(t,t_0) = U_S(t,t_0)U_B(t,t_0) = \exp\left\{-\frac{i}{\hbar}H_S(t-t_0)\right\}\exp\left\{-\frac{i}{\hbar}H_B(t-t_0)\right\}. \tag{37}$$

This leads to a new equation of motion in a form

$$\frac{\partial}{\partial t}\bar{W}^{(n)}(t) = -i\mathcal{L}_I(t)\bar{W}^{(n)}(t) + \bar{S}^{(n)}(t), \tag{38}$$

where we defined a Liouville superoperator $\mathcal{L}_I(t)$ corresponding to the interaction Hamiltonian in interaction picture (denoted by $H_I(t)$):

$$\mathcal{L}_I(t)A = \frac{1}{\hbar}\left[H_I(t), A\right]_- . \tag{39}$$

Here, A is an arbitrary operator, and we defined a source term in interaction picture

$$\bar{S}^{(n)}(t) = U_0(t_0,t)S^{(n)}(t)U_0(t,t_0), \tag{40}$$

with

$$S^{(n)}(t) = \frac{i}{\hbar}\left[\mu, W^{(n-1)}(t)\right]_- E(t). \tag{41}$$

We will now proceed along the lines of the standard projection operator technique (see e.g. [7, 8]) by defining two projection superoperators $\mathcal{P}$ and $\mathcal{Q}$ (acting on the operators $W^{(n)}(t)$) such that

$$\mathcal{P} + \mathcal{Q} = 1. \tag{42}$$

In particular we will use the well-known Argyres-Kelley projection superoperator which acts on $W^{(n)}(t)$ as

$$\mathcal{P}W^{(n)}(t) = \mathrm{tr}_{\mathrm{bath}}\left\{W^{(n)}\right\} w_{\mathrm{eq}}, \tag{43}$$

where w_{eq} is the bath equilibrium density matrix. Applying these operators to Eq. (38) and eliminating $\mathcal{Q}W^{(n)}(t)$ we obtain a variant of the well-known Nakajima-Zwanzig identity:

$$\frac{\partial}{\partial t}\mathcal{P}\bar{W}^{(n)}(t) = -i\mathcal{P}\mathcal{L}_I(t)\mathcal{P}\bar{W}^{(n)}(t) + \mathcal{P}\bar{S}^{(n)}(t) - i\mathcal{P}\mathcal{L}_I(t)\mathcal{U}_{\mathcal{Q}}(t,t_0)\mathcal{Q}\bar{W}^{(n)}(t_0)$$

$$-i\int_{t_0}^{t} \mathrm{d}\tau \mathcal{P}\mathcal{L}_I(t)\mathcal{U}_{\mathcal{Q}}(t,\tau)\mathcal{Q}\bar{S}^{(n)}(\tau) - \int_{t_0}^{t} \mathrm{d}\tau \mathcal{P}\mathcal{L}_I(t)\mathcal{U}_{\mathcal{Q}}(t,\tau)\mathcal{Q}\mathcal{L}_I(\tau)\mathcal{P}\bar{W}^{(n)}(\tau). \tag{44}$$

Here,

$$\mathcal{U}_{\mathcal{Q}}(t,\tau) = \exp_{\rightarrow}\left\{-i\int_{\tau}^{t} \mathrm{d}z\mathcal{Q}\mathcal{L}_I(z)\mathcal{Q}\right\}. \tag{45}$$

The first term on the right hand side (r.h.s.) in Eq. (44) can be eliminated by an appropriate choice of the form of interaction operator H_I. We assume that the system-bath interaction operator is of a pure dephasing form in the basis of the electronic states localized on the molecules of the complex (the site basis)

$$H_I = \sum_n \Delta V_n |n\rangle\langle n|. \tag{46}$$

We can always redefine this interaction operator in such a way that

$$\mathcal{P}H_I = 0 \tag{47}$$

by setting $H_I' = H_I - \mathcal{P}H_I$. The Hamiltonian operators H_I and H_I' differ only by a shift of the excitation energies local to the involved molecules, the so-called reorganization energies. The reorganization energies can be always made part of the system Hamiltonian. We assume further on that Eq. (47) holds.

The second term on the r. h. s. of Eq. (44) is a source term by which the operator of nth order of perturbation is connected to the lower order operator. The third term is the so-called initial correlation term. Since the initial condition is always $W^{(n)}(t_0) = 0$ (and t_0 is a time before any interaction with the field), this term also disappears. The fourth term has a very similar structure as the initial correlation term. It underlines the fact that the true initial condition for the $W^{(n)}$ is set by the source term, and, in the case that some correlation between the bath and the system is created during the process of excitation, it contributes to the excited state evolution.

The last term of Eq. (44) is responsible for the processes of energy relaxation and transfer and for dephasing of coherences. We will treat this term in line with Ref. [7] to turn it into a second order time local differential equation. We define formally a superoperator $\bar{\mathcal{U}}(t,t_0)$ which describes an exact evolution of the projected statistical operator $\mathcal{P}\bar{W}^{(n)}(t)$. In Eq. (44) we substitute $\mathcal{P}\bar{W}^{(n)}(\tau) = \bar{\mathcal{U}}(\tau,t)\mathcal{P}\bar{W}^{(n)}(t)$. Now $\mathcal{P}\bar{W}^{(n)}(t)$ can be taken out of the integral and the differential equation is local in time. Next we will approximate the integral by a second order expression in terms of $\mathcal{L}_I(t)$. Both $\bar{\mathcal{U}}(\tau,t)$ and $\mathcal{U}_{\mathcal{Q}}(t,\tau)$ contain all orders of $\mathcal{L}_I(t)$, however, in order to keep the integral in second order, we only need to use their zero order approximations $\bar{\mathcal{U}}(\tau,t) \approx 1$ and $\mathcal{U}_{\mathcal{Q}}(t,\tau) \approx 1$. Using the second order approximation in the last term of Eq. (44) we get a precursor to a derivation of the standard Redfield relaxation equations. We notice that all terms start with $\mathcal{P}$ and correspondingly, we perform the trace in all terms and divide out the equilibrium bath density matrix w_{eq}. The result reads as

$$\frac{\partial}{\partial t}\bar{\rho}^{(n)}(t) = \bar{s}^{(n)}(t) + \bar{I}^{(n)}(t,t_0) - \int\limits_{t_0}^{t} \mathrm{d}\tau \ \mathrm{tr}_{\mathrm{bath}} \left\{\mathcal{L}_I(t)\mathcal{Q}\mathcal{L}_I(\tau)w_{\mathrm{eq}}\right\} \bar{\rho}^{(n)}(t), \tag{48}$$

where we defined a reduced source terms

$$\bar{s}^{(n)}(t) = \mathrm{tr}_{\mathrm{bath}} \left\{ \bar{S}^{(n)}(t) \right\}, \tag{49}$$

and

$$\bar{I}^{(n)}(t, t_0) = -i \int_{t_0}^{t} \mathrm{d}\tau \; \mathrm{tr}_{\mathrm{bath}} \left\{ \mathcal{L}_I(t) \mathcal{U}_{\mathcal{Q}}(t,\tau) \mathcal{Q} \bar{S}^{(n)}(\tau) \right\} \tag{50}$$

is a photo-induced correlation term. All terms in Eq. (48) are in interaction picture with respect to the system Hamiltonian H_S (this is denoted by the bar). The effect of interaction picture with respect to H_B vanishes, because under the trace over the bath DOF we can rotate the arguments and eliminate the corresponding evolution operators. Eq. (48) equation can be turned back to Schrödinger picture in a straightforward way. In the following section we will apply Eq. (48) to evaluate $\rho^{(1)}(t)$ and $\rho^{(2)}(t)$.

C. First Order in Field

The first order reduced density matrix is driven by the first order source term $s^{(1)}$which contains the zero order density matrix

$$S^{(1)}(t) = \frac{i}{\hbar} [\mu, W^{(0)}(t)]_{-} E(t), \tag{51}$$

where

$$W^{(0)}(t) = w_{\mathrm{eq}} |g\rangle\langle g|. \tag{52}$$

Here we already returned to Schrödinger picture. The equilibrium density matrix of the system does not evolve in time, and, correspondingly, the only time evolution in the source term originates from the electric field. As a consequence one can easily perform the trace to obtain $s^{(1)}(t)$ in a form

$$s^{(1)}(t) = \frac{i}{\hbar} \sum_{k} \mu_{kg} e^{i\omega_{kg}(t-t_0)} E(t) |k\rangle\langle g| + \mathrm{h.c.}, \tag{53}$$

where h.c. stands for Hermite conjugate, and $\mu_{kg} = \langle k|\mu|g\rangle$ is the transition dipole moment between the ground state $|g\rangle$ and an excited state $|k\rangle$. One can easily show that $\mathcal{Q}\bar{S}^{(1)}(t) = 0$, and thus the first order equations of motion reads as

$$\frac{\partial}{\partial t} \rho^{(1)}(t) = -\frac{i}{\hbar} \left[H_M, \rho^{(1)}(t) \right]_{-} + s^{(1)}(t) - \mathcal{R}^{(2)}(t, t_0) \rho^{(1)}(t), \tag{54}$$

where

$$\mathcal{R}^{(2)}(t,t_0) = \int_{t_0}^{t} \mathrm{d}\tau \ \mathrm{tr}_{\mathrm{bath}} \left\{\mathcal{L}_I(t)\mathcal{Q}\mathcal{L}_I(\tau)w_{\mathrm{eq}}\right\} \tag{55}$$

is a relaxation tensor of a second order in system–bath interaction term.

For an infinite bath with a unstructured spectral density coupled weakly to our system, the integrand in Eq. (55) decays rather quickly with a certain characteristic time t_c (termed bath correlation time). The integral in Eq. (55) becomes constant on the same time scale. In Eq. (54) we have a rather arbitrary constant t_0 representing some time at which we start to treat the dynamics. In the full equation with $n = 1$ before reduction, i.e. in Eq. (34), of which Eq. (54) is an approximation, setting t_0 to any value such that $E(t_0) = 0$ leads to the same correct result. However, in Eq. (54) the choice of t_0 does affect the dynamics. We could always choose t_0 sufficiently small to make the relaxation tensor constant. For pulses longer than the bath correlation time, even if we set the start of the dynamics, t_0, before the center of the pulse so that $E(t_0) < \epsilon$ where ϵ is some chosen small value, the relaxation tensor will integrate into constant already during the action of the pulse. This is a consequence of the decoupling of the bath from the influence by external field which is otherwise indirectly mediated by the electronic DOF. This decoupling is a consequence of reducing our equations of motion to electronic DOF only. The only point in which the bath state is influenced in our description is the initial condition. Eq. (54) can therefore describe the dynamics correctly only if the excitation field is in a form of the Dirac delta function centered at some τ'. Than the start of the dynamics can be set to $t_0 = \tau'$, and the bath can correctly react to the excitation of the system. One can demonstrate this idea by using Eq. (33) to obtain $\rho^{(1)}(t)$ - this procedure involves no additional approximation. We obtain

$$\rho^{(1)}(t) = \int_{t_0}^{t} \mathrm{d}\tau \ \left[J(t,\tau) + J^{\dagger}(t,\tau)\right] E(\tau), \tag{56}$$

where

$$J(t,\tau) = \frac{i}{\hbar}\mathrm{tr}_{\mathrm{bath}} \left\{U(t,\tau)\mu|g\rangle\langle g|w_{\mathrm{eq}}U(\tau,t)\right\}, \tag{57}$$

is an operator on the same Hilbert space as $\rho^{(1)}(t)$. Eq. (57) can be written as

$$J(t,\tau) = \mathcal{U}(t,\tau)\mu\rho_0, \tag{58}$$

where $\rho_0 = |g\rangle\langle g|$, and $\mathcal{U}(t,\tau)$ is a formally exact evolution superoperator for the reduced density matrix. Eq. (56) corresponds to a sum (integral) of field free evolutions starting

from an initial condition $[\mu, \rho_0]_-$ at times τ weighted by the field amplitude $E(\tau)$. The exact field free evolution has to involve an exact time dependent relaxation tensor $\mathcal{R}(t, \tau)$. Notice that the second argument coincides with the start of the dynamics for a given field free propagation.

The discussion above underlines the fact that the RDM master equation, Eq. (54), can only be correct if the source term is of a Dirac delta form, or if the transient features of the relaxation tensor are unimportant. It turns out that for the first order density matrix $\rho^{(1)}(t)$ and its subsequent application to calculation of the second order RDM $\rho^{(2)}$, the early dynamics is important. If we used Eq. (54) with constant relaxation (dephasing) tensor, we would limit the lineshape of the transitions to Lorentzian type. For more general and realistic lineshapes we need to use Eq. (56).

D. Secular Approximation in First Order Equations

For the first order term in the field, the transient features of the relaxation tensor turn out to be of crucial importance. They are responsible for the line shape of linear absorption. The absorption spectrum of a multilevel molecular system can be calculated as (see e.g. [9])

$$\alpha(\omega) \approx 2\mathrm{Re} \int_0^\infty \mathrm{d}t\ \langle g|\mu\mathcal{U}(t,0)\mu\rho_0|g\rangle e^{i\omega t}. \tag{59}$$

Here the initial condition $\mu\rho_0$ which consists entirely of optical coherences is propagated in time. In general the evolutions of different optical coherences are coupled. Nevertheless, the so-called secular approximation in which decoupling of individual coherences is postulated is very often valid. In particular, it can be shown that secular approximation is strictly valid in second order for a homodimer [10]. For molecules with similar reorganization energies, non-secular effects are very small, and only for systems were the participating states have very different reorganization energies one can find pronounced effects of coupled evolution of different optical coherences [11]. Because our interest in this work is with photosynthetic systems composed of pigments of similar kind (chlorophylls and bacteriochlorophylls) we can expect the secular approximation to work reasonably well. We can therefore assume that only the elements

$$J_{ag}(t, \tau) = \langle a|J(t, \tau)|g\rangle = \mathcal{U}_{agag}(t, \tau)\mu_{ag} \tag{60}$$

of the operator, Eq. (57) are non-zero. In secular approximation, Eq. (54) splits into a set of independent equations for coherences. Green's function corresponding to the secular version of Eq. (54) gives a second order approximation to $\mathcal{U}_{agag}(t,\tau)$. It is possible to show that for a bath composed of harmonic oscillators, second order master equation for an optical coherence gives an exact result [12]. It can be further shown that

$$\mathcal{U}_{agag}(t,\tau) = e^{-g_{aa}(t-\tau)-i\omega_{ag}(t-\tau)}, \tag{61}$$

and

$$\mathcal{R}_{agag}(t,\tau) = \frac{\partial}{\partial t} g_{ag}(t-\tau) \tag{62}$$

with

$$g_{aa}(t) = \frac{1}{\hbar^2}\int\limits_0^t \mathrm{d}\tau \int\limits_0^\tau \mathrm{d}\tau' \; \mathrm{tr}_{\mathrm{bath}} \left\{ \Delta V_{aa}(\tau)\Delta V_{aa} w_{\mathrm{eq}} \right\}, \tag{63}$$

representing a lineshape function of the transition $|g\rangle \rightarrow |a\rangle$. For details see e.g. [8].

To conclude this section, in secular approximation we can obtain the reduced density matrix elements of the first order in field to all orders of the perturbation in the system-bath coupling. This is a very good starting point for the derivation of the source term for the second order.

E. Photo-induced Initial Correlation Term in Second Order Equations

Second order field perturbation of the density matrix enables us to calculate populations of excited states relevant for SMS. In the experiment, we observe fluorescence from excited states populated due to relaxation dynamics subsequent to the excitation by external field. According to Eq. (41), the second order source term has a form

$$s^{(2)}(t) = \frac{i}{\hbar}\left[\mu, \rho^{(1)}(t)\right]_{-} E(t). \tag{64}$$

This result is general, i.e. independent of our previous discussion of secular approximation. We have discussed the validity of secular approximation for first order equations in preceding section. The validity of the secular approximation in the second order term, however, has to be discussed separately. In the excited state band, non-secular terms can fine tune the final, quasi-equilibrium (electronically excited) state of the system reached after the propagation (see e.g. [9]). If secular approximation is not valid, the coherences do not decay to zero.

If, however, the working basis is chosen well for the particular problem, e.g. delocalized excitonic basis is chosen in case of weak system-bath interaction, or localized basis is chosen for strong system-bath coupling, the need for non-secular theory can be minimized. Most importantly, large body of theoretical understanding of photosynthesis is based on the secular approximation. We will therefore apply secular approximation in second order equations to simplify our treatment.

Unlike in the case of the first order RDM, second order contribution $\mathcal{Q}S^{(2)}(\tau)$, which enters the photo-induced initial correlation term (see Eq. (50)), is in general non-zero. Before we derive the second order reduced equations of motion in Section IV F of this SI, we will treat below this additional term.

We are interested in a contribution of the photo-induced initial correlation term to populations of excited states, and we will therefore express this contribution in the basis of the eigenstates of the electronic Hamiltonian. For populations we can keep working in interaction picture

$$\bar{I}^{(n)}_{aa}(t,t_0) = \langle a|I^{(n)}(t,t_0)|a\rangle$$
$$\approx \frac{2}{\hbar}\int\limits_{t_0}^{t} \mathrm{d}\tau \sum_b \mathrm{Im}\ \mathrm{tr}_{\mathrm{bath}}\left\{H^I_{ab}(t)\langle b|\mathcal{Q}\bar{S}^{(n)}(\tau)|a\rangle\right\}, \tag{65}$$

where we made the same approximation as in the relaxation tensor, namely, that $\mathcal{U}_Q(t,\tau) \approx 1$. The term $\mathcal{P}\bar{S}^{(n)}(\tau)$ which is a part of the $\mathcal{Q}\bar{S}^{(n)}(\tau) = (1-\mathcal{P})\bar{S}^{(n)}(\tau)$ term is by definition of $\mathcal{P}$ proportional to w_{eq} and $\mathrm{tr}_{\mathrm{bath}}\left\{H^I_{ab}(t)w_{eq}\right\} = 0$ because of Eq. (47). The $\mathcal{Q}$ projector is therefore effectively equal to unity, here. Using the system-bath interaction Hamiltonian elements in eigenstate basis

$$\Delta V_{ab}(t,t_0) = \sum_n \langle a|n\rangle\langle n|b\rangle\Delta V_n(t,t_0), \tag{66}$$

we can write the pulse correlation term as

$$\bar{I}^{(n)}_{aa}(t) = \frac{2}{\hbar}\int\limits_{t_0}^{t} \mathrm{d}\tau \sum_b \mathrm{Im}\ \mathrm{tr}_{\mathrm{bath}}\left\{\Delta V_{ab}(t)\langle b|\bar{S}^{(n)}(\tau)|a\rangle\right\}. \tag{67}$$

A straightforward but tedious derivation using second order cumulant expansion and rotating wave approximation leads to an expression in terms of bath correlation functions

$$C_{ab,cd}(t) = \frac{1}{\hbar^2}\mathrm{tr}_{\mathrm{bath}}\left\{\Delta V_{ab}(t)\Delta V_{cd}(0)w_{\mathrm{eq}}\right\}, \tag{68}$$

and the corresponding line shape functions

$$g_{ab,cd}(t) = \int_{t_0}^{t} \mathrm{d}\tau \int_{t_0}^{\tau} \mathrm{d}\tau' C_{ab,cd}(\tau'). \tag{69}$$

The expression reads as

$$I_{aa}(t,t_0) = \sum_b \frac{d_{ag}d_{gb}}{\hbar^2} \int_{t_0}^{t} \mathrm{d}\tau \ \mathrm{Im}\ e^{i\omega_{ab}(t-\tau)} K_{ab}(t,\tau), \tag{70}$$

where

$$K_{ab}(t,\tau) = i\hbar \Big[\int_{t_0}^{\tau} \mathrm{d}\tau' \mathcal{E}(\tau)\mathcal{E}^*(\tau') e^{-i(\Omega-\omega_{ag})(\tau-\tau')} \int_0^{\tau-\tau'} \mathrm{d}\tau'' C^*_{ab,aa}(t-\tau+\tau'') e^{-g^*_{aa,aa}(\tau-\tau')}$$

$$- \int_{t_0}^{\tau} \mathrm{d}\tau' \mathcal{E}(\tau)\mathcal{E}^*(\tau') e^{i(\Omega-\omega_{bg})(\tau-\tau')} \int_0^{\tau-\tau'} \mathrm{d}\tau'' C_{ab,bb}(t-\tau+\tau'') e^{\cdot -g(\tau-\tau')} \Big]. \tag{71}$$

It is easy to show that $K_{aa}(t,\tau) = 0$ and therefore all contributions to the population from the pulse induced correlation term are of the non-secular form. The light excites coherence between states $|a\rangle$ and $|b\rangle$ and the term of the first order in system bath interaction ($\Delta V_{ab}(t)$) is responsible for converting this contribution to a population of state $|a\rangle$. All terms K_{ab} for $a \neq b$ oscillate. Therefore, we can ignore the contribution of $\bar{I}_{aa}$ to the populations in our work for the same reason we ignore other non-secular contributions. For slow field envelops $\mathcal{E}(t)$ and resonant pulses (the case in SMS) the term $\bar{I}_{aa}(t,t_0)$ contains an integral over oscillating functions and it is itself oscillating. Contributions from different times therefore cancel and the overall contribution to populations is small.

Besides the smallness of the contribution due to oscillation, one can find situations where $\bar{I}_{aa}$ yields zero for other reasons. Because K_{ab} is proportional to correlation functions

$$C_{ab,bb}(t) = \sum_n \langle a|n\rangle\langle n|b\rangle |\langle b|n\rangle|^2 C_{nn}(t), \tag{72}$$

we can see that K_{ab} is zero if the delocalized states $|a\rangle$ and $|b\rangle$ do not share the same sites. For instance, when the excitonic basis coincides with the site basis (vanishing coupling between sites) the term $\bar{I}_{aa}$ is automatically zero. Another case yielding zero is a case of homodimer. There we can assume that $g_{aa,aa}(t)$ and $g_{bb,bb}(t)$ are the same, and we can also assume that

$$C_{ab,bb}(t) = C(t) \sum_n \langle a|n\rangle\langle n|b\rangle |\langle b|n\rangle|^2 = \xi_{ab,bb} C(t), \tag{73}$$

where the bath correlation function $C(t) \equiv C_n(t)$ is the same for all (both) sites. The second equals sign in Eq. (73) defines the coefficients $\xi_{ab,bb}$. The term K_{ab} then consists of two terms proportional to $(\xi_{ab,aa} - \xi_{ab,bb})$ and $(\xi_{ab,aa} + \xi_{ab,aa})$, respectively. These terms are both zero. For a homodimer $(\xi_{ab,aa} + \xi_{ab,aa}) = \langle a|b\rangle = 0$ for $a \neq b$, and $(\xi_{ab,aa} - \xi_{ab,bb}) = 0$ because all terms $|\langle a|n\rangle|^2$ are equal to $\frac{1}{2}$.

F. Second Order Equations of Motion

Similar to Section IV C, we can use Eqs. (33) or (34) to derive either a closed equation for the second order RDM $\rho^{(2)}(t)$ or a corresponding differential equation of motion. To obtain the closed equation based on Eq. (33) we would have to derive a time evolution superoperator, in analogy to Eq. (58), for all possible initial times τ. This amounts to propagating the differential equation derived from Eq. (34) for every time τ separately, with a source term corresponding to a Dirac delta pulse centered at time $t = \tau$. As in Section IV C, we cannot reproduce an exact result for $\rho^{(2)}(t)$ with a single propagation of a RDM equation of motion even, if we were in possession of a exact relaxation tensor $\mathcal{R}(t,\tau)$. Nevertheless, because populations change on a much slower time scale than coherences, and because we are in fact only interested in populations at long times after excitation, a constant relaxation tensor obtained by the limit $\mathcal{R}_{\text{eff}}(t) = \lim_{t_0 \to -\infty} \mathcal{R}(t,t_0)$ is expected to be a reasonable approximation for the description of our SMS experiment. The effective relaxation tensor does not depend on the initial condition time, but it still weakly depends on the running time of the experiment (as discussed in the main text). Neglecting the photo-induced initial correlation term, we obtain the following equations of motion for the second order RDM:

$$\frac{\partial}{\partial t}\rho^{(2)}(t) = -\frac{i}{\hbar}\left[H_M, \rho^{(2)}(t)\right]_- + s^{(2)}(t) - \mathcal{R}^{(2)}_{\text{eff}}(t)\rho^{(2)}(t). \tag{74}$$

Applying secular approximation, writing out explicit form of the source term $s^{(2)}(t)$, and selecting only equations for eigenstate populations, we arrive at Eq. (8) of the main text.

[1] Novoderezhkin, V. I., Palacios, M. A., van Amerongen, H. & van Grondelle, R. Energy-Transfer Dynamics in the LHCII Complex of Higher Plants: Modified Redfield Approach. *J. Phys. Chem. B* **108**, 10363 (2004).

[2] Mukamel, S. *Principles of nonlinear spectroscopy* (Oxford University Press, Oxford, 1995).

[3] Krüger, T. P. J., Novoderezhkin, V. I., Ilioaia, C. & van Grondelle, R. Fluorescence spectral dynamics of single LHCII trimers. *Biophys. J.* **98**, 3093–101 (2010).

[4] Valkunas, L., Chmeliov, J., Krüger, T. P. J., Ilioaia, C. & van Grondelle, R. How Photosynthetic Proteins Switch. *J. Phys. Chem. Lett.* **3**, 2779–2784 (2012).

[5] Chmeliov, J., Valkunas, L., Krüger, T. P. J., Ilioaia, C. & van Grondelle, R. Fluorescence blinking of single major light-harvesting complexes. *New J. Phys.* **15**, 85007 (2013).

[6] van Kampen, N. G. *Stochastic processes in physics and chemistry* (North Holland, 1992).

[7] Fain, B. *Irreversibilities in Quantum Mechanics* (Kluer Acadenic Publishers, Dorderecht, 2000).

[8] Valkunas, L., Abramavicius, D. & Mančal, T. *Molecular Excitation Dynamics and Relaxation: Quantum Theory and Spectroscopy* (WILEY-VCH Verlag, Berlin, 2013).

[9] Olšina, J., Mančal, T., Olsina, J. & Mancal, T. Electronic Coherence Dephasing in Excitonic Molecular Complexes: Role of Markov and Secular Approximations. *J. Mol. Model.* **16**, 1765 (2010).

[10] Mančal, T. *et al.* Electronic coherence transfer in photosynthetic complexes and its signatures in optical spectroscopy. *Spectroscopy* **22**, 199–211 (2008).

[11] Olšina, J., Kramer, T., Kreisbeck, C. & Mančal, T. Exact stochastic unraveling of an optical coherence dynamics by cumulant expansion. *The Journal of chemical physics* **141**, 164109 (2014).

[12] Doll, R., Zueco, D., Wubs, M., Kohler, S. & Hänggi, P. On the conundrum of deriving exact solutions from approximate master equations. *Chemical Physics* **347**, 243–249 (2008).
